# Accelerometry in the Functional Assessment of Balance in People with Stroke: A Systematic Review

**DOI:** 10.3390/jcm12247701

**Published:** 2023-12-15

**Authors:** Juan Francisco Pérez-López, Roberto Cano-de-la-Cuerda, Rosa María Ortiz-Gutiérrez

**Affiliations:** 1 Department of Physical Therapy, Occupational Therapy, Rehabilitation and Physical Medicine, Faculty of Health Sciences, Universidad Rey Juan Carlos, 28922 Madrid, Spain; jf.perez@alumnos.urjc.es; 2 Radiology, Rehabilitation and Physiotherapy Department, Nursing, Physiotherapy and Podiatry Faculty, Complutense of Madrid University, Plaza Ramón y Cajal 3, 28040 Madrid, Spain; rosaorti@ucm.es

**Keywords:** accelerometer, balance, functional assessment, stroke, reliability, validity

## Abstract

Balance disturbances in people with lived experience of stroke affect activities of daily living and social participation, so assessing them is essential to know the level of functional independence. Accelerometers are electronic devices that allow kinematic variables of balance to be recorded and are a tool of great interest in the assessment of functional balance. To determine the validity and reliability of, as well as the most performed protocols using accelerometers in the functional assessment of balance in people with experience of stroke, a systematic search of articles published in the electronic databases PubMed, Scopus, the Web of Science, the Cochrane Library, the PEDro and the Virtual Health Library from Spain was performed following the PRISMA (Preferred Reporting Items for Systematic Reviews and Meta-Analysis) guidelines. We used QUADAS-2 to assess the quality of the included studies. Eight studies met the inclusion criteria, two studied reliability and validity, two studied reliability and four studied the validity of accelerometers in the assessment of balance in people with stroke. All studies indicated the kind of accelerometer, localization on the body, tests and outcome variables. The results indicate that accelerometers show excellent reliability values in the assessment of balance in people who had a prior stroke and disparate results in terms of validity. Triaxial accelerometers were most used, and the 4th and 5th lumbar and 1st and 2nd sacral vertebrae were the body areas most used for their placement.

## 1. Introduction

Stroke is defined by the World Health Organization (WHO) as ‘a clinical syndrome of rapid development due to a focal disturbance of cerebral function of vascular origin and lasting more than 24 h’. It is the second leading cause of death in the world population and one of the main causes of disability in adults [1,2,3]. Among the clinical signs that most impact functional capacity and quality of life, balance is one of the aspects most affected after suffering a stroke [4].

A total of 78% of people with lived experience of stroke have balance disorders, mainly caused by somatosensory and muscle tone impairment [5]. These balance disturbances produce limitations in their ability to stand and walk and increase the risk of falls. This limits the performance of activities of daily living and constitutes an accessibility barrier for the economic and social performance of people with experience of stroke [6,7].

Balance is a strong predictor of the level of functional independence in the evolution of stroke, hence the importance of carrying out a correct assessment [8]. For the functional assessment of balance in clinical and research settings, healthcare professionals have a wide range of validated clinical scales, as well as instrumental systems [9]. On the one hand, the clinical scales are easy to use, quick to administer and do not require expensive equipment. However, their limitations include dependence on subjective aspects related to the evaluator, such as experience in their administration and the possible interpretation of the results [9]. On the contrary, instrumental systems, including posturography, are the reference systems for assessing balance due to their high degree of precision and reliability [10]. However, these systems are expensive, not very portable and require specialized technical experience, which makes their implementation in clinical practice difficult [11].

In this context, of the instrumental systems for balance assessment, accelerometers are electronic devices that record the angular velocity and linear acceleration of the displacement of the different body segments from which kinematic parameters such as the orientation, position, speed, posture and range of motion of the joints can be determined [12]. The use of these devices, which first focused on monitoring physical activity, dates to the 1980s. However, in the last decade, and thanks to the miniaturization of electronic components of computer and mobile phone systems, their use in functional assessment has been widespread [13].

Accelerometers are characterized by being portable, low cost and having few restrictions on the types of movements they can monitor [14]. Triaxial accelerometers are the most widely used nowadays, and they can be placed in different parts of the body, the hip, wrist and thigh being the most common areas in clinical research [15]. Further, these devices can be used to evaluate different domains of balance not only in the laboratory but also in situations of the person’s daily life [15].

Accelerometry has been more widely used as a system for measuring functional capacity in relation to the maintenance of balance in individuals with lived experience of stroke compared with other neurological disorders [16]. Unlike observational clinical scales, balance assessment using accelerometers can provide more information about body displacement in different tasks and conditions of instability at a more affordable cost and in a more accessible way than using laboratory systems [17].

Two of the qualities to consider when assessing the efficacy of accelerometry as a measurement instrument are validity (the degree of confidence we can have that the measurement corresponds to the reality of the phenomenon being measured) and reliability (measurement precision). Considering the volume of scientific literature published in the last decade about accelerometers, the aim of this research was to carry out a systematic review of the psychometric properties (validity and reliability), the most performed protocols and the usefulness of accelerometry systems in the assessment of balance in people with lived experience of stroke.

## 2. Methods

A systematic review of articles published in electronic databases was carried out, following the Preferred Reporting Items for Systematic Reviews and Meta-Analyses (PRISMA) instructions for conducting systematic reviews [18]. In addition, the protocol of this paper was registered in Systematic Review Data Repository Plus (SRDR+) (protocol registration no. SR-360).

Between March and April 2022, two independent reviewers (JFPL, RMOG) searched the electronic databases PubMed, Scopus, the Web of Science (WOS), the Cochrane Library, the Physiotherapy Evidence Database (PEDro) and the Virtual Health Library from Spain (BVS). The search strategies used are presented in Table 1.

The following inclusion criteria were established: (a) observational studies (longitudinal and cross-sectional studies); (b) people of both sexes with a confirmed diagnosis of stroke; (c) use of an accelerometer system for the functional assessment of balance; and (d) papers published between 2012 and 2022.

Search results were uploaded to the bibliographical citation manager Zotero, and duplicates were removed. Afterward, two independent reviewers completed an Excel template with the title and abstract of all uploaded citations to analyze. The order in which the documents were eliminated was: (1) non-observational design; (2) did not include people with experience of stroke; (3) did not use accelerometry; and (4) non-assessment of balance. In the event of uncertainty, a third reviewer moderated the process until a consensus was reached. 

The articles selected were obtained in full text and were evaluated by the research team to determine their relevance to the review aims.

Data was extracted into prepared Excel tables by two independent reviewers and was verified by a third reviewer for accuracy when discrepancies were present. These tables detail the author, year and country of study, participants, a description of the accelerometer used, how they were used (alone or combined with other scales) and the main results in the assessment of balance in people with experience of stroke. 

All studies that recorded a variable of accelerometry, including linear and angular acceleration, linear and angular velocity and displacement distance, were considered for the selection of results, regardless of the technological device in which the accelerometers were integrated. This way, studies that used accelerometry sensors, inertial systems and smartphones were included to assess balance in people with experience of stroke.

The quality of the included studies was analyzed by two independent reviewers using the QUADAS-2 [19] instrument, reaching an agreement when there were discrepancies in the evaluation. This scale contains four domains: patient recruitment, the index test, the reference standard and the flow of patients through the study and the moments at which the index and reference tests were performed (flow and times). In turn, in each domain, the probability of bias and applicability were assessed, qualifying them as ‘low’, ‘high’ or ‘uncertain’. 

## 3. Results

**Figure 1:**
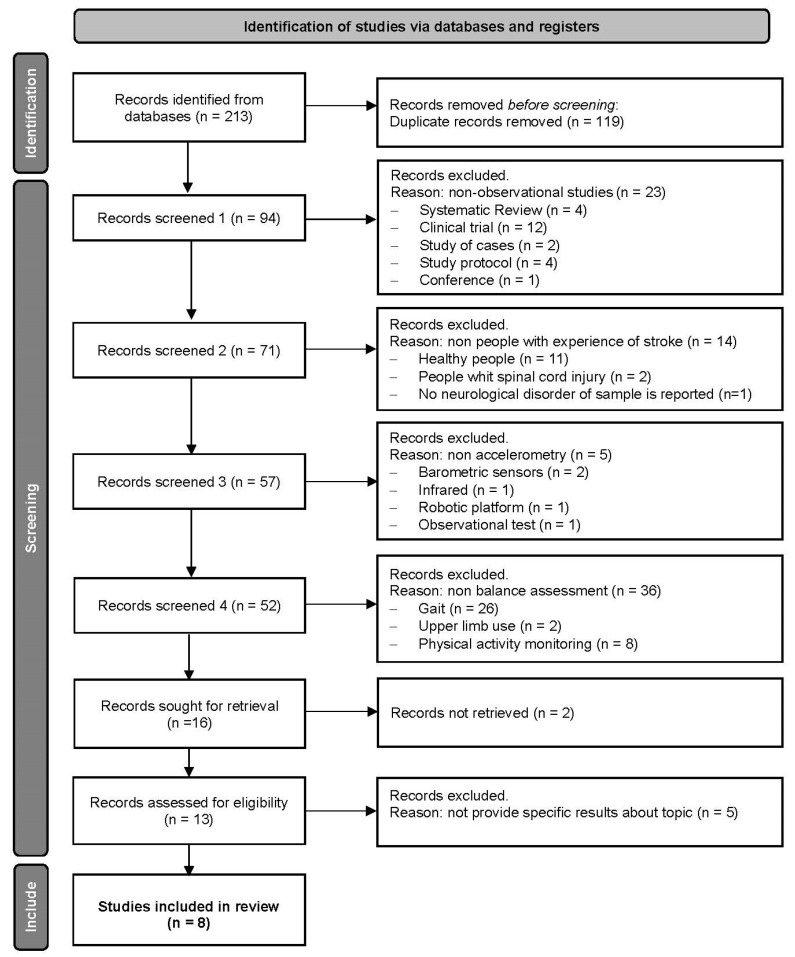
PRISMA Flow Diagram [20]. All records were excluded by a human.

After applying the search equations in the databases, a total of 213 studies were retrieved. After eliminating duplicates, 94 were recovered. Subsequently, after reading the title and abstract, 12 studies were selected for complete reading. Four were excluded according to the eligibility criteria. Finally, eight studies were selected for the present systematic review. Figure 1 shows the search algorithm and screening process of the studies based on the PRISMA Flow Diagram [20].

### 3.1. Study Design

The studies included in the review were published between 2014 and 2019. All studies were of the observational type, two of them being cross-sectional. Regarding the psychometric properties of accelerometers, in two studies, the authors indicated that the purpose was to evaluate reliability [21,22], although one studied validity [22]. Three studies analyzed reliability and validity [23,24,25], two studies only analyzed validity [26,27] and one study did not specifically state a purpose [28]. 

In relation to validity, five studies employed a case-control design (the performance of healthy subjects was compared with that of people with experienced stroke in balance tests) [22,24,25,27,28], and three studies used a convergent validity design (analyzed the concordance between measures) [23,25,26] to study the criteria validity of accelerometers. As for reliability, one study assessed the test-retest reliability of accelerometers [24] and two assessed intra- and inter-observer reliability in a sample of people with lived experience of stroke [21,23]. In one study, the reliability test was in healthy adults; therefore, these results were not considered for this review [25].

In all of the studies, the authors explained the inclusion criteria but did not cite the method of recruitment or sampling. Four studies presented the approval of an ethics committee and informed consent of the patient and indicated that they complied with the ethical standards of the Declaration of Helsinki [21,23,26], while one mentioned only the approval of the committee [27].

### 3.2. Participants

The demographic characteristics of participants were generally well documented in each study. We observed variations among studies in some variables, including sample sizes that ranged from four to five participants [21,23] to 30 [28]; ages between 18 and over 65 years; the course of disease that was reported in four studies [21,22,28]; three studies that recruited people with experience chronic stroke and in one with sub-acute stroke [27]; and the etiology of the stroke cited in two studies, with 22 ischemic and 5 hemorrhagic strokes and 22 ischemic and 8 hemorrhagic strokes, respectively [27,28].

### 3.3. Balance Evaluation Methods

Among the different types of accelerometers used in balance assessment, two studies used smartphone accelerometers [22,25], two used inertial measurement units (IMU) [24,27], and the other four used triaxial accelerometers [21,23,26,28].

The accelerometer placement protocol varied in each study depending on the number of sensors used, which ranged from one to eight. The body areas where the accelerometers were placed included the 5th lumbar [28], the 2nd sacral vertebrae [22,25] and a combination of sensors on the 7th thoracic 5th lumbar and 1st sacral vertebrae [21,23], the occipital bone, sternum, 4th and 5th lumbar vertebrae and external malleolus [27]; and wrists, shanks, 3rd lumbar vertebrae and feet [24].

Accelerometer recordings were simultaneously combined with validated balance tests for stroke, such as the Functional Reach Test (FRT) [23], the Single-Leg Stance Test (SLS) [21], the Clinical Test of Sensory Interaction and Balance (CTSIB) [26], the Timed Up and Go Test (TUG) [24,28] and the 50-step version of the Fukuda Stepping Test (FST) [27]. Moreover, in two studies to determine validity, data obtained by the accelerometers were compared with the results of the Berg Balance Scale (BBS) [25,26]. In two studies, they used accelerometers during a specifically designed protocol consisting of the maintenance of six postures that compromised standing balance, such as standing with eyes closed and open, a different base of support width, and tandem and semi-tandem positions [22,25].

### 3.4. Outcome Variables

In relation to static balance, the accelerometers recorded the maximum angular lumbosacral/thoracic displacement and time of displacement during the performance of FRT [23], maximum displacement and maximum velocity of displacement of the trunk relative to movements in each axis (*x*, *y*, *z*) during the SLS [21], and acceleration of the displacement of the center of mass in different sensory conflict conditions [26] and at different velocities [22,25]. One study recorded a set of indices related to body accelerations during the performance of the FST (root mean square, attenuation coefficients and improved harmonic ratio) [27].

Finally, two studies used accelerometers as an instrument for recording balance variables during sit-to-stand and stand-to-sit during TUG tests. One study recorded the duration and maximum angular trunk velocity of the sit-to-walk transition [24], whereas another study recorded the acceleration of the center of mass [28].

### 3.5. Signal Processing

Regarding the signal processing of the sensors used in the included studies, in one study, the authors indicated that they used Kalman filtering algorithms to calculate displacement, velocity and resultant [21]. In two studies, data were processed using custom algorithms implemented in the MATLAB software version 9.14 (The MathWorks Inc., Natick, MA, USA) [24,27]. In another study, the EMGworks program (Delsys, Inc., Boston, MA, USA) was used to convert the values of acceleration to Root Mean Square (RMS) values [26]. In another study, the accelerometer data were transferred to a laptop computer and analyzed using BTS G-studio software version 2.6.12.0 (BTS Bioengineering S.p.A., Aradeo, Italy) [28]. In the case of studies that used a smartphone as an accelerometer, the information provided by its built-in accelerometer and gyroscope was recorded with SensorKinects Pro (INNOVENTIONS Incorporation, Houston, TX, USA), an application that collects sensor data from smartphones [22,25]. In only one study, the authors indicated the mathematical formulas used to calculate the variables but not the signal processing system [23]. 

Regarding the formulas used for signal processing, in four studies, the authors converted each acceleration component (j) to RMS values [X(rms) = sqrt (x12 + x22 + … + xn2)] and normalized RMS values by dividing the RMS of records at each axis and body level [23,26,27]. 

Moreover, in two studies, the authors calculated Attenuation Coefficients (AC) for each acceleration component (j), which represents the variation in acceleration from lower to upper body levels defined by the formula: [AC Upper-Lower-Body level = 1 − (RMS Upper-Body level/RMS Lower-Body level)]. These authors also calculated the improved harmonic ratio (iHR) for each acceleration component (j) by the algorithm: [∑ amplitude intrinsic harmonics)/∑ amplitude extrinsic harmonics]. In this formula, intrinsic harmonic characterizes the ideal balance and extrinsic deviations from the ideal balance [27]. In one study, together with RMS, signal vector magnitude (SMV) was calculated by the formula [SMV= sqrt (x2 + y2 + z2)] [26]. 

In one study, the outcome variables were cited (displacement and velocity), but only the formula used to calculate the Resultant velocity (Rv) was reported, expressed as [Rv= sqrt (Vx2 + Vy2 + Vz2)] [21]. The authors of another study do not report the application of a specific formula for signal processing, recording the unit of measurement of the acceleration range of the variables in m/s^2^ [28].

On the other hand, in one of the included studies, the authors referred to a previous study in which the authors filtered signals (θg–lp; atrunk–lp) to determine the trunk posture during transitions from sitting to standing and non-transitions posture [24]. They calculated the acceleration vector using parameters: Range (θg–lp), Min (θg–lp), Range (atrunk–lp), Min (atrunk–lp), Max (atrunk–lp), t {Min (atrunk–lp) and t {Max (atrunk–lp).

Finally, from smartphone accelerometry data, the combined changes in the acceleration vector from the anterior-poster and medial-lateral axes to represent the postural control ability were calculated. It calculated by algorithm [ [(sqrt (x2 − x1)2 + (y2 − y1)2] + [(sqrt (x3 − x2)2 + (y3 − y2)2] … [(sqrt (xn − xn)2 + (yn − y2)2]/n − 1] [22,25].

### 3.6. Result of Studies

Among the studies that analyzed the validity of accelerometers, one study found statistically significant differences between people with lived experience of stroke and healthy individuals (*p* < 0.005). The time of test performance variable showed that in eight TUG parameters, lower values were observed in people with a prior stroke [24]. In the same line, the results of another study showed that the durations of sit-to-stand and stand-to-sit transitions during TUGs were significantly longer for the patients with experience of stroke than for the healthy subjects (*p* < 0.05). Also, the acceleration ranges of the body were slower in patients with a prior stroke (*p* < 0.01) during the sit-to-stand but not during the stand-to-sit phase (*p* > 0.05) [28]. 

Another study found a significant correlation (*p* < 0.05) between mediolateral and anteroposterior displacements detected by a triaxial accelerometer in all CTSIB conditions and in the BBS result [26]. On the other hand, one study found statistically significant differences (*p* = 0.001) in body sway recordings detected by accelerometers of a smartphone between people with stroke and healthy subjects in the maintenance of four of the six postures utilized to assess balance [22]. 

In contrast, another study found no statistically significant differences between healthy and subjects with a prior stroke in the maintenance of balance in the six postures that were studied (*p* = 0.07–0.65) [25]. In addition, the convergent validity test did not show a significant correlation between the accelerometer measurements of smartphones and the BBS (*p* = 0.053 and *p* = 0.723) [25]. Similarly, the results of another study did not show statistically significant differences in the magnitude of rotation and anteroposterior and mediolateral displacements of the body detected by IMU in the FST between people with experience of stroke and healthy persons [27]. 

As for the studies that analyzed reliability, one showed excellent test–retest reliability (intraclass correlation coefficient, ICC = 0.855–0.994) in the measurements of an inertial sensor in 12 variables of the 14 TUG metrics analyzed [24]. On the other hand, the results of another study showed excellent intra- and interobserver reliability with ICC values above 0.85 for the displacement and velocity variables detected by the triaxial accelerometers in the SLS test [21].

Regarding the location of the accelerometers, the results of one study showed no statistically significant differences between the recordings of accelerometers placed in the dorsal and lumbar region during the SLS, concluding that the location of the sensors is not relevant when recording the maximum displacement of the trunk movements [21]. In contrast, in another study, differences were observed in the maximum trunk displacement in the FRT between the data provided by the sensor placed in the lumbar region with respect to the dorsally placed sensor [23].

The summary of the main results of each of the articles included in the review is shown in Table 2.

### 3.7. Risk of Bias Results

Overall, in relation to bias probability, the QUADAS-2 results indicated a high probability of risk in the selection of participants since recruitment and selection processes were not specified in any of the studies. We rated the risk probability in relation to the domain of the index test as uncertain because the authors of the studies did not provide clear information in this regard. On the other hand, the studies did not include a reference standard test, so we considered the probability of bias in this domain to be high. However, we have considered the flow and time domains to have a low probability of bias for most of the studies since the tests for the assessment of balance were performed with a minimum time interval between them [21,22,24,25,26,27,28].

Finally, regarding the applicability of results, in the domain of the standard reference test, three studies used reference tests with good psychometric properties to assess balance [23,25,28], so we considered the concern to be low. For the rest of the studies, we considered the concern to be high since the assessment of balance did not include this reference test.

**Figure 2:**
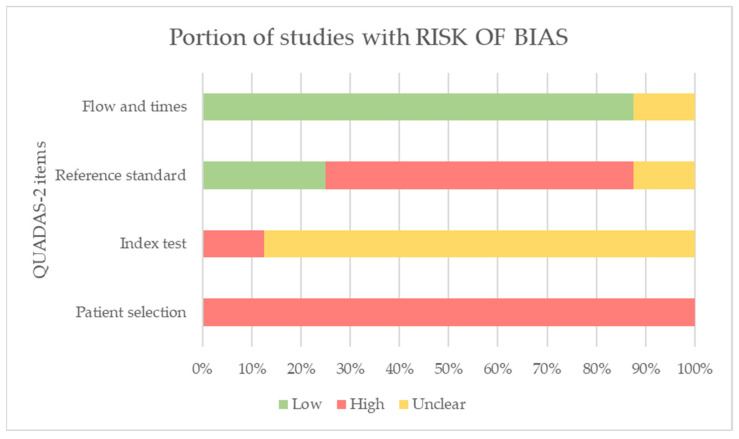

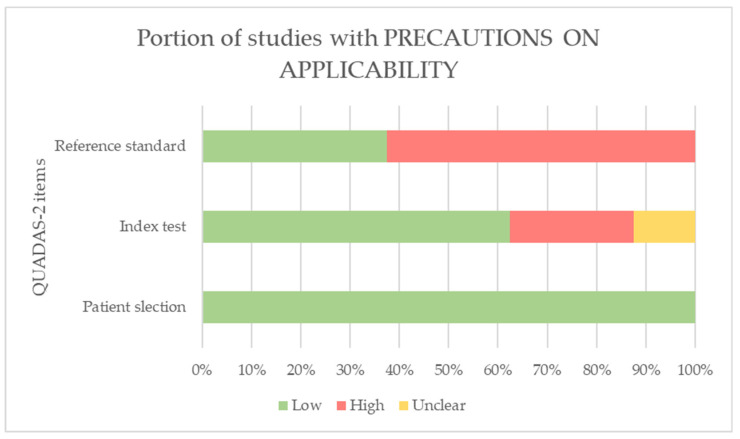
QUADAS-2 graphics.

The results of the QUADAS-2 domains for each of the included studies are shown in Table 3. Figure 2 show the graphs of the QUADAS-2 results.

## 4. Discussion

The aim of this systematic review was to understand the current evidence about the application of accelerometry systems in assessing balance in people who have suffered a stroke. Of the 155 studies that were previously compiled, 8 studies were included after the corresponding screening process [21,22,23,24,25,26,27,28]. This objective is of great interest since, in recent years, the wireless systems most used in balance assessment have been accelerometers and gyroscopes [29]. Accelerometry can provide data to aid this assessment [16].

In general, the included studies aimed to determine the validity and reliability of different accelerometry devices as instruments for recording variables related to balance in patients with experience of stroke, both in situations that require stability in maintaining standing and in dynamic conditions of position change. In this regard, the systematic review of Bruyneel et al. [30] indicates that balance assessment tests with a dynamic component in patients with a prior stroke show better psychometric properties than those that only assess balance in the maintenance of standing. However, in five of the included studies, the accelerometers were used during static balance tests, such as the FRT [23], SLS [21] and CTSIB [26], as well as in specific protocols whose tests required the maintenance of different postures in situations of sensory conflict [22,25]. In three studies, the accelerometers were used under dynamic balance conditions, such as sit-to-stand and stand-to-sit transition (TUG) [24,28] or with modification of the base of support (FST) [27].

Recent studies support the incorporation of accelerometry devices in functional balance assessment by validated clinical scales for the accuracy of measurements, which seems comparable to that of posturography systems, as well as their low cost in relation to these instrumental systems [17]. The studies included in this review used accelerometers to record variables of the linear acceleration of different body segments [27] and the displacement of the center of mass [22,23,25], the main study variable of posturography systems [9]. In this manner, the use of validated clinical scales in combination with accelerometers reduces observer-dependent biases. None of the studies included in this review used a posturography system as the reference balance test to analyze the validity of accelerometers.

As for the results of the studies, all those that analyzed the reliability of accelerometers for functional balance assessment in people with stroke showed values considered excellent. Nevertheless, in the studies that analyzed validity, the results showed disparate values in the correlation of the accelerometry systems with clinical scales that measure the same construct, as well as in the performance between healthy and stroke participants in the assessment of balance. This may be due to different evaluation protocols in terms of the type of devices used, their placement, the signal processing and the clinical scale of reference used for each of the studies. 

In this sense, the results of the present review are in line with the conclusions of a recent systematic review about the diagnostic capacity of accelerometers in fall risk assessment in people with chronic stroke, whose authors pointed to limited evidence because no uniformity existed in the literature on placement, number of accelerometers or type [31]. Therefore, future studies are required to establish the sensitivity and specificity of accelerometers in the assessment of balance [31].

Referring to the presence of bias, the studies included in this review correspond to observational study designs and only two of them defined the temporality as cross-sectional [21,23]. A single study used simple blinding on the evaluators for data extraction of the variables [23].

The quality of most of the included studies is considered low, mainly due to the procedures for patient selection and evaluator and patient blinding, as well as the absence of a reference test. In this sense, small sample sizes were used without specifying the participant sampling and selection processes. In addition, there is a lack of information regarding the sample characteristics and assessment protocols, which impacts the reproducibility of the studies. Similarly, no studies specified the process for recruiting and assigning participants, which may affect the internal validity of the studies. Furthermore, participants with mild to moderate alterations in physical condition and adequate cognitive ability to follow instructions were included, so the studies’ external validity could be equally limited.

Similarly, this review suffers from some limitations, such as the impossibility of including two studies that were of interest, not being able to access their full text through the distribution platforms of scientific publications, or contacting their corresponding authors. Otherwise, although the initial search found numerous publications in which accelerometry is used in the functional assessment of patients with stroke, these studies did not indicate aspects regarding the validity and reliability of these systems, so they were discarded.

In future lines of research, given that the ability to maintain balance is a strong clinical predictor in the prognosis of patients in the early stages after suffering a stroke [32], it would be interesting to be able to establish criteria based on accelerometry parameters that identify the value of balance prediction.

## 5. Conclusions

The assessment of balance in patients with lived experience of stroke using triaxial accelerometers has been widely used, with the 4th–5th lumbar and 1st–2nd sacral vertebrae being the body areas most used for placement. According to the literature reviewed, the recording of balance variables through accelerometers has been carried out in combination with validated tests in the context of stroke. Lastly, the data observed indicate excellent reliability in the use of accelerometers as instruments for assessing balance in people with experience of stroke, although it was not possible to obtain conclusive data regarding the validity of these devices due to the existence of disparate results. 

## Figures and Tables

**Table 1:** Search strategies used in electronic databases.

**PUBMED**	**RESULTS**
#1 “Accelerometry” [MeSH]	11,296
#2 “Accelerometry” [Title/Abstract]	5351
#3 “Acelerom *” [Title/Abstract]	20,923
#4 #1 OR #2 OR #3	25,657
#5 “Stroke” [MeSH]	158,593
#6 “Stroke” [Title/Abstract]	282,951
#7 #5 OR #6	322,672
#8 “Outcome and Process Assessment, Health Care” [MeSH]	1,311,980
#9 “Assessment”	1,602,783
#10 #8 OR #9	2,648,945
#11 “Postural Balance” [MeSH]	26,759
#12 “Balance” [Title/Abstract]	254,645
#13 #11 OR #12	266,277
#14 #4 AND #7 AND #10 AND #13	28
#15 Limit #14 TO: published in the last 10 years	21
Total PUBMED results	21
**SCOPUS**	**RESULTS**
#1 “Accelerom *” [Title-Abstract-Keywords]	70,031
#2 “Stroke” [Title-Abstract-Keywords]	482,261
#3 “Balance” [Title-Abstract-Keywords]	876,153
#4 #1 AND #2 AND #3	75
#5 Limit #4 TO: published in the last 10 years	60
Total SCOPUS results	60
**WEB OF SCIENCE**	**RESULTS**
#1 “Accelerom *” [Topic]	52,046
#2 “Stroke” [Topic]	405,619
#3 “Balance” [Topic]	620,526
#4 #1 AND #2 AND #3	90
#5 Refined #4 By: Publication Years: 2022 or 2021 or 2020 or 2019 or 2018 or 2017 or 2016 or 2015 or 2014 or 2013	69
Total WOS results	69
**COCHRANE LIBRARY**	**RESULTS**
#1 “Accelerometry” [MeSH descriptor]	1075
#2 “Accelerom *”	1
#3 #1 OR #2	1076
#4 “Stroke” [MeSH descriptor]	11,217
#5 “Stroke”	75,606
#6 #4 OR #5	75,901
#7 “Postural Balance” [MeSH descriptor]	3120
#8 “Balance”	30,553
#9 #7 OR #8	30,555
#10 #3 AND #6 AND #9	3
#11 Limit #10 TO: published in the last 10 years	3
Total, COCHRANE LIBRARY results	3
**PEDro**	**RESULTS**
#1 Accelerom * Stroke Balance	2
Total PEDro results	2
**BVS Spain**	**RESULTS**
Stroke AND Accelerom * AND Balance	58
Total, BVS results	58

BVS: Virtual Health Library from Spain; MeSH: Medical Subject Headings; PEDro: The Physiotherapy Evidence Database.

**Table 2:** Results of the systematic review.

Author and Year	Population (Women/Men)	Accelerometer Type	Accelerometer Location	Variable Measured	Psychometric Properties of the Accelerometer Analyzed	Results
Hou et al. (2019) [25]	19	Accelerometer of the ASUS Zenfone 3 smartphone	S2 vertebra	Displacement of the center of gravity in the base of support	Reliability and validity of a proprietary scale and comparison BBS	In the reliability test, in which healthy subjects participated, the intraclass correlation coefficient (ICC) of the accelerometer was 0.904 within-day and 0.764 between-days, indicating excellent reliability. In the validity test, in the accelerometer data, no statistically significant differences were observed between healthy subjects and subjects with stroke in the six tests (*p* = 0.007–0.65). The criterion validity test did not show significant correlations between the accelerometer and the BBS, obtaining *p*-values between 0.053 and 0.723.
Hou et al. (2018) [22]	23 (9/14)	Accelerometer of the HTC 10 smartphone	S2 vertebra	Changes in acceleration of the center of gravity at the base of support	Validity of a proprietary scale	Significant differences were found between stroke subjects and healthy subjects in four test postures, with *p*-values between 0.000 and 0.048.
Belluscio et al. (2018) [27]	18 (12/6)	Opal (IMU) (APDM Inc., Portland, OR, USA)	Occipital, sternum, L4–L5 vertebrae and both external malleoli	Linear accelerations (ML, AP and rotation) during the FST	Validity during the FST	No significant differences were found in the magnitude of body rotation and AP/ML displacement between stroke patients classified 3–4 in the FAC and healthy people, showing that the FST parameters are not capable of distinguishing between healthy and pathological subjects.
Na et al. (2016) [28]	60 (30/30)	Tri-axial accelerometer (G-Walk; BTS Bioengineering S.p.A., Italy)	L5 vertebra	Phase duration (s), AP, ML and VP acceleration range (m/s^2^),	Validity during the sit-to-stand and stand-to-sit transition of TUG	significant differences (*p* < 0.05) between patients with stroke and healthy subjects were found in the phase duration and the AP, ML, and VT acceleration ranges sit-to-stand. And in duration m (*p* < 0.05) and ML acceleration range (*p* < 0.001) stand-to-sit and phase.
Wüest et al. (2016) [24]	39 (19/20)	Physilog (GaitUp, Lausanne, Switzerland)	Both wrists, both legs, thoracic spine, both feet and L3 vertebra	Total time, sit-to-gait transfer, gait characteristics, turn, turn-to-sit transfer	Reliability and validity during the sit-to-stand and stand-to-sit transition of TUG	In the reliability test, of the 14 TUG metrics analyzed, 12 variables showed excellent test–retest reliability (ICC = 0.855–0.994). Regarding validity and taking time into consideration, there was a statistically significant difference between the two groups (*p* = 0.002). Of the 13 parameters analyzed, 8 showed a significant difference between groups (*p* = 0.000–0.02). In addition, 11 of the 14 parameters analyzed showed a low standard error of measurement and low minimum detectable difference.
Chung et al. (2016) [26]	27 (6/21)	Trigno™ Wireless Electromyography System (Delsys Inc., Boston, MA, USA)		Postural sway	Validity during the CTSIB and a comparison with BBS	A significant correlation (*p* < 0.05) was found between the left–right and forward–backward accelerometry measures of the CTSIB with the BBS scores. Moreover, it showed a significant correlation between the acceleration of condition 3 of the CTSIB and the total score of the BBS (correlates *p* < 0.05).
Pérez-Cruzado et al. (2014) [21]	4	InertiaCube3TM (InterSense Inc., Bedford, MA, USA)	T7–T8 and L5–S1 vertebrae	Variables of movement and speed of the trunk in rotation, flexion/extension and inclination	Reliability during the SLS	The ICC showed values over 0.847 for all the variables, both interobserver and intraobserver in both devices. Therefore, reliability showed excellent values for displacement and speed. Furthermore, significant differences in location were only found in 2 of the 68 variables measured, so the location of the sensors for the SLS between the upper trunk and the lower back would not be relevant.
Merchán-Baeza et al. (2014) [23]	4	InertiaCube3TM (InterSense Inc., Bedford, MA, USA)	L5–S1 and T7 vertebrae	Variables of trunk displacement and time	Reliability during the FRT	The within-subject reliability values observed in the use of inertial sensors were all above 0.820 (ICC = 0.829–0.891). The observed between-subject ICC values ranged from 0.821 to 0.883. Therefore, the intra- and intersubject reliability could be categorized as excellent. On the other hand, the intersubject and intrasubject reliability of the FRT was 0.987 (0.983–0.992) and 0.983 (0.979–0.989), respectively, being excellent in both cases. The levels of reliability observed could be categorized as excellent based on the results for intraobserver reliability (ICC = 0.829–0.878) and interobserver reliability (ICC = 0.821–0.883). In addition, it showed differences between the data provided by the sensors in the lower back with respect to the rest of the trunk when performing the FRT.

10MWT: 10-Meter Walk Test; AP: antero-posterior; BBS: Berg Balance Scale; BI: Barthel Index; ICC; intraclass correlation coefficient; CTSIB: Clinical Test of Sensory Interaction and Balance; FAC: Functional Ambulation Classification scale; FRT: Functional Reach Test; FST: Fukuda Stepping Test; IMU: inertial measurement unit; ML: medio-lateral; SLS: Single-Leg Stance Test; TUG: Timed Up and Go.

**Table 3:** Summary of QUADAS-2 results for risk of bias and applicability of included studies.

Authors	Bias Probability				Concerns Regarding Applicability of Results		
	Patient Selection	Index Test	Reference Standard	Flow and Times	Patient Selection	Index Test	Reference Standard
Pérez-Cruzado et al. [21]	High	Unclear	High	Low	Low	Low	High
Hou et al. [22]	High	Unclear	High	Low	Low	Unclear	High
Merchán-Baeza et al. [23]	High	Unclear	High	Low	Low	Low	High
Wüest et al. [24]	High	Unclear	High	Low	Low	Low	High
Hou et al. [25]	High	Unclear	Low	Low	Low	High	Low
Chung et al. [26]	High	Unclear	Unclear	Unclear	Low	Low	Low
Belluscio et al. [27]	High	Unclear	High	Low	Low	High	High
Na et al. [28]	High	Unclear	Low	Low	Low	Low	Low

## Data Availability

Not applicable.

## Abbreviations

10MWT	10-Meter Walk Test
AP	Antero-posterior
BBS	Berg Balance Scale
BI	Barthel Index
BVS	Virtual Health Library from Spain
CTSIB	Clinical Test of Sensory Interaction and Balance
FAC	Functional Ambulation Classification scale
FRT	Functional Reach Test
FST	Fukuda Stepping Test
ICC	Intraclass correlation coefficient
IMU	Inertial measurement units
MeSH	Medical Subject Headings
ML	Medio-lateral
PEDro	Physiotherapy Evidence Database
PRISMA	Preferred Reporting Items for Systematic Reviews and Meta-Analysis
RMS	Root Mean Square
SLS	Sigl-Leg Stance Test
TUG	Timed Up and Go Test
WHO	World Health Organization
WOS	Web of Science
